# Discrepancy in alloy composition of imported and non-imported porcelain-fused-to-metal (PFM) crowns produced by Norwegian dental laboratories

**DOI:** 10.1080/26415275.2020.1724512

**Published:** 2020-02-11

**Authors:** Håvard Jostein Haugen, Brandon Michael Soltvedt, Peter N. Nguyen, Hans Jacob Ronold, Gaute Floer Johnsen

**Affiliations:** a Department of Biomaterials, Institute of Clinical Dentistry, Faculty of Dentistry, University of Oslo, Oslo, Norway; b Department of Prosthetic, Institute of Clinical Dentistry, Faculty of Dentistry, University of Oslo, Oslo, Norway

**Keywords:** Dental crown, metal ceramic alloys, porcelain-metal alloys, metal ceramic restorations

## Abstract

**Purpose:**

Even though the use of full ceramic crowns have become a well-established practice in dental clinics compare to the last decade, the use of imported casted porcelain-fused-to-metal (PFMs) crowns is still prevalent. The use of imported PFMs is often economically driven; however, when dentists order PFMs, they do not have capabilities to examine its true alloy content. Therefore, we raise the questions whether cheaper imported PFMs have more discrepancies in alloy content compared to domestically produced PFMs?

**Materials and Methods:**

This study included 62 porcelain-fused-to-metal crowns: 41 produced in Norway and 21 imported. Their alloy-composition was determined non-destructively by EDX and SEM.

**Results and Conclusions:**

Imported PFMs demonstrated larger deviations compared with non-imported PFMs. Significant deviation was found in key metallic elements in the different alloys (W, In, Pd, Ag). The detected deviations in key element such as Wolfram and Indium could influence the PFMs service time. These finding may be of international concern

## Introduction

Porcelain fused to metal crowns (PFM) provide an opportunity to restore damaged, nonfunctional teeth back to their proper function and esthetics. These restorations have been among the most prevalent fixed prosthodontic restorations over the last 50 years. PFMs have a long history of clinical success and have been considered the gold standard. Studies have shown long-term survival rates (>8 years) between 92.3–95.9% [1]. The traditional alloys were gold-based noble alloys and the majority of the long-term survival rate studies of PFMs are based on these compositions. However, due to the fluctuating price of palladium and other noble metals, the use of these alloys has decreased over the past decade [2]. This decline has given rise to the use of alternative alloys, but also on an increase of imported PFMs [3,4]. A survey from 2009 showed that 71% of the questioned Norwegian dentists used imported prosthetic restorations in their practices [5]. It is impossible for a dentist to verify the actual alloy content of prosthetic materials.

The motivation behind this study was a growing concern over discrepancies of imported and nonimported produced dental restorations [4]. Only a few studies outside of Scandinavia have conducted similar compositional analyses on imported restorations. These studies included a low numbers of restorations and employed destructive methods making follow-up studies of the clinical performance impossible [4,6]. A study by the Swedish Medical Products Agency (Läkemedelsverket) in 2010 found that 46% (6 out of 13) of imported gold alloys and 69% (9 out of 13) of imported base metal alloys differed significantly from the specific alloy ordered [7]. The number of compositional discrepancies for the domestic orders was 8% (1 out of 13) for gold alloys and 31% (4 out of 13) for base metal alloys. Swedish study from 2011 compared fixed dental prostheses (FDP) produced in Sweden and China and found that 85% (11 out of 14) the imported FDP did not contain the specified gold alloy [8]. The findings from the aforementioned studies bolsters concerns regarding the quality of imported PFMs and bolsters concerns regarding the quality of imported PFMs. The previous studies comprised a limited number of restorations and applied destructive methods of analysis, which precluded future clinical follow-up of the PFMs. Furthermore, prior studies have used fictitious patients [4,8] and the clinical outcome of the reported compositional discrepancies could not be tested. In this study, a larger number of restorations than in previous studies have been analyzed.

The primary purpose of this study was to determine the alloy composition of imported and non-imported PFMs (single crowns) and to compare the determined alloy compositions with those claimed by the dental laboratories to arrive at the level of compositional discrepancy. The secondary aim was to examine whether the delivered PFMs complied with the Declaration of Conformity. Our null hypothesis was that there would be no difference in the level of compositional discrepancy between imported and non-imported PFMs.

## Materials and methods

The study included 62 PFMs: 41 produced in Norway and 21 imported. The crowns were randomly selected from crowns ordered by undergraduate dental students to patients receiving treatment at the Institute for Clinical Dentistry, Faculty of Dentistry, University of Oslo. The crowns were delivered by seven different local dental laboratories, which had no knowledge of any testing. Imported crowns were produced by the respective laboratories´ subcontractors located abroad. The patients were not included in the study, as the focus of this study was the alloy content of the PFMS constructions. Therefore, no judgments were made in terms of aesthetics (anatomical contours, color, polish, etc.) or general workmanship of the outer porcelain veneer.

Standard procedure at the Institute was followed for ordering crowns: impressions in elastomeric material (Impregium Permadyne Penta, 3 M ESPE), quality controlled by experienced and calibrated instructors and forwarded to the respective laboratories alongside standardized ordering forms with specifications of color, shape, type of alloy/materials and other relevant information.

All available crowns with a metal–alloy substructure were accepted as specimens for this study. This was the sole criterion for inclusion of crowns. There were no set exclusion criteria. The alloys included in the study were: (1) Co–Cr (predominantly base metal): CopraBond K, Cara SLM, Kera^®^-disc, Remanium 2001, Wirobond SG, Wirobond 280 2) Ag–Pd (noble metal 1): Argelite 61, d.SIGN^®^53, and 3) Au–Pd–Ag (noble metal 2): (See Tables 1 and 2 for composition).

**Table 1.** Elementary analysis in weight percentage (wt. %) for nonprecious alloys (CoCr), *CopraBond K, Cara SLM, Kera-disc, Remanium 2001, Wirobond SG and Wirobond 280.*

NON-PRECIOUS ALLOYS	Co	Cr	W	Mo	Mn	Fe	Si	Ga
CopraBond K	61.0	28.0	8.5		0.3	<0.5	1.7	
Cara SLM	61.80–65.8	23.7–25.7	4.9–5.9	4.60–5.60	<0.1	<0.5	<1.2	
Kera^®^-disc	61.7	27.8	8.5		0.3	0.2	1.6	
Remanium 2001	63.0	23.0	4.3	7.3	<1.0		1.6	
Wirobond SG	63.8	24.8	5.3	5.1		✓	✓	
Wirobond 280	60.2	25.00	6.2	4.8	✓		✓	2.9

*Remanium 2001 also contains trace amounts of Nitrogen (N).* Symbol (✓): Element stated to be present in unspecified trace amount according to manufacturer. Empty box: amount below detection limit.

**Table 2.** Precious alloys, *d.SIGN 53, Argelite 61, Precious 1 and Precious 2.*

PRECIOUS ALLOYS	Pd	Ag	Sn	In	Ru	Re	Li	Pt	Au	Ga
d.SIGN^®^53	53.8	34.9	7.7	1.7	<1.0	<1.0	<1.0	<1.0		
Argelite 61	60.6	28.1	2.5	6.6	<1.0	<1.0				2.1
Precious 1	61.4	26.0	6.0	4.0	0.1					
Precious 2	53.7	35.7		1.0					1.0	

Amounts stated in weight percentage (wt.%). Empty box: amount below detection limit.

Alloy-composition was determined by energy-dispersive X-ray spectroscopy in a Hitachi Analytical Table Top Microscope/Benchtop SEM TM3030 (EDX-SEM) (Hitachi, Japan). The method of examination was nondestructive. The metal content of the alloys was presented as weight percent (wt.%). The crowns were placed on the specimen stage in the chamber of the EDX-SEM machine before the chamber was placed in a vacuumed state prior to line analysis measurements at three randomly chosen areas. A levelled and homogenous site on the metal alloys surface was analyzed for 60 s. The quantification of element B5 to Am95 in accordance to the periodic table was performed, and with particular attention to nickel (Ni) and cadmium (Cd), in accordance to ISO-standard 22674: 2006 Dentistry – Metallic materials for fixed and removable restorations and appliances [9].

The seven dental laboratories were designated Laboratory A, B, C, D, E, F and G to maintain anonymity. After analysis, each crown was numbered chronologically for identification. Results for each crown were reviewed and contaminating elements (e.g. oxygen O)) were excluded in order to give a more representative proportion of the alloy constituents. PFMs with large deviations in alloy-content were analyzed at two different sites to confirm findings.

Subsequently, the crowns were grouped according to laboratories, alloy-type and organized in predetermined categories defined as: *No deviation* included crowns with only minor deviations in major constituents (elements that comprised >20 wt. %) and slight deviations in additional elements that comprised <10 wt. %. *Small deviation* included crowns with deviations <5 wt. percentage concerning major constituents and/or deviations >1 wt. % in additional elements that comprised <10 wt. %. *Large deviation* included deviations >5wt. % concerning major constituents and missing additional elements. This category also included crowns with elements that should not be found in the relevant alloys such as aluminum (Al) or were not specified. Incorrect alloy refers to crowns that comprised of elements similar to a different type of alloy. For example, a crown marked as CopraBond K with results showing elements such as silver (Ag) and palladium (Pd) and is likely an Argelite61 alloy. Unspecified alloy were crowns delivered without or with lacking alloy information.

Three categories of alloy specification based on reoccurring styles of enclosed documents was defined: *Specified content* refers to crowns provided with adequate information, either as a specified alloy (e.g. CopraBond K) or as a general alloy enclosed with a further specification of the alloy composition, thereby enabling analysis comparison. Several crowns were labelled as ‘Noble metal’; however, the technician specified the alloy content in detail. These were termed ‘Noble metal 1’ and ‘Noble metal 2’ as these two variants were reoccurring. *No content information* refers to crowns delivered without information or crowns only provided with a general metal alloy and not further specified (such as CoCr, but not further specified). Crowns delivered with such labelling are defined as label X in the Supplementary Tables S1–S4 found in the appendix. *Incorrect information* refers to crowns delivered with content different from the information provided by the laboratories. Crowns labelled as CoCr or Noble metal, but analysis indicated otherwise are defined as label Z or content *.

Statistical analyses were conducted with SigmaPlot 13 (Systat Software, IL, USA). All datasets were tested for normality (Shapiro–Wilk) and equal variance to determined nonparametric or parametric behavior. Two-tailed one sample t-test was used to compare parametric datasets. One-Sample Signed Rank Test was used for nonparametric dataset. All groups which had a sample mean exceeding the hypothesized mean by an amount that is greater than would be expected by chance, are labelled with a hashtag (#) (Figure 2, Table S1–S4). Statistical significance was set at a level of 0.05.

## Results

**Figure 1.**
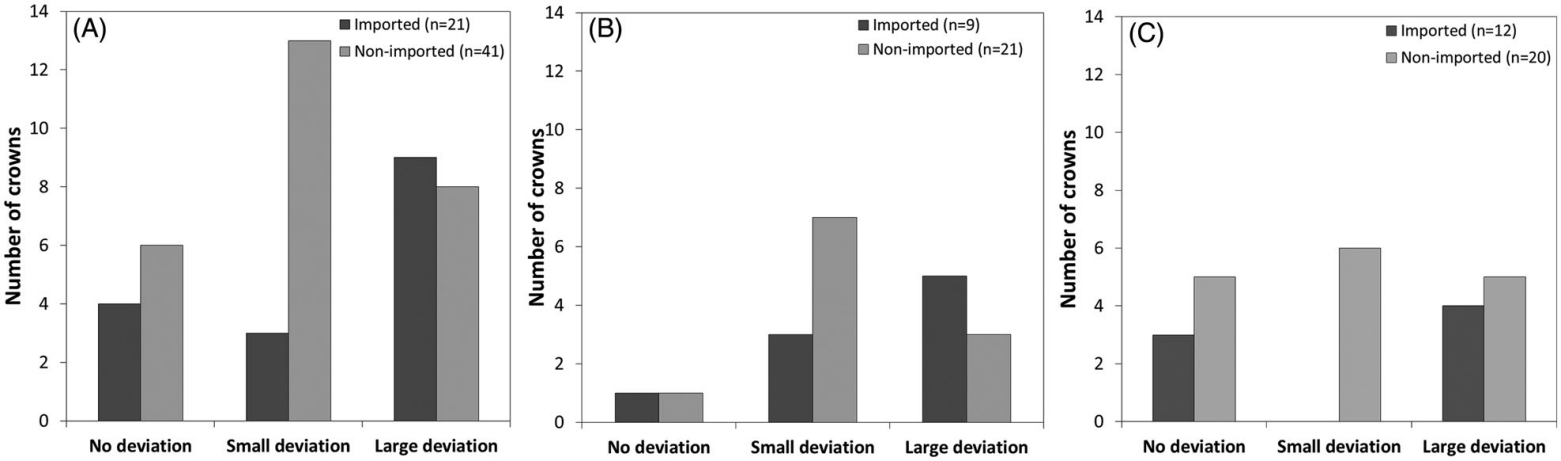
Number of base-metal crowns sorted in categories according to degree of deviation (A: all base metals, B: Predominantly base metal base metal only C: noble metal base metal only.) No deviation: Minor deviations in major constituents (elements that comprises >20 wt. %) and slight deviations in additional elements that comprise <10 wt.%. Small deviation: <5 wt. % concerning major constituents and/or deviations >1 wt. % in additional elements that comprise <10 wt.%. Large deviation: >5wt. % concerning major constituents and missing and/or additional elements.

**Figure 2.**
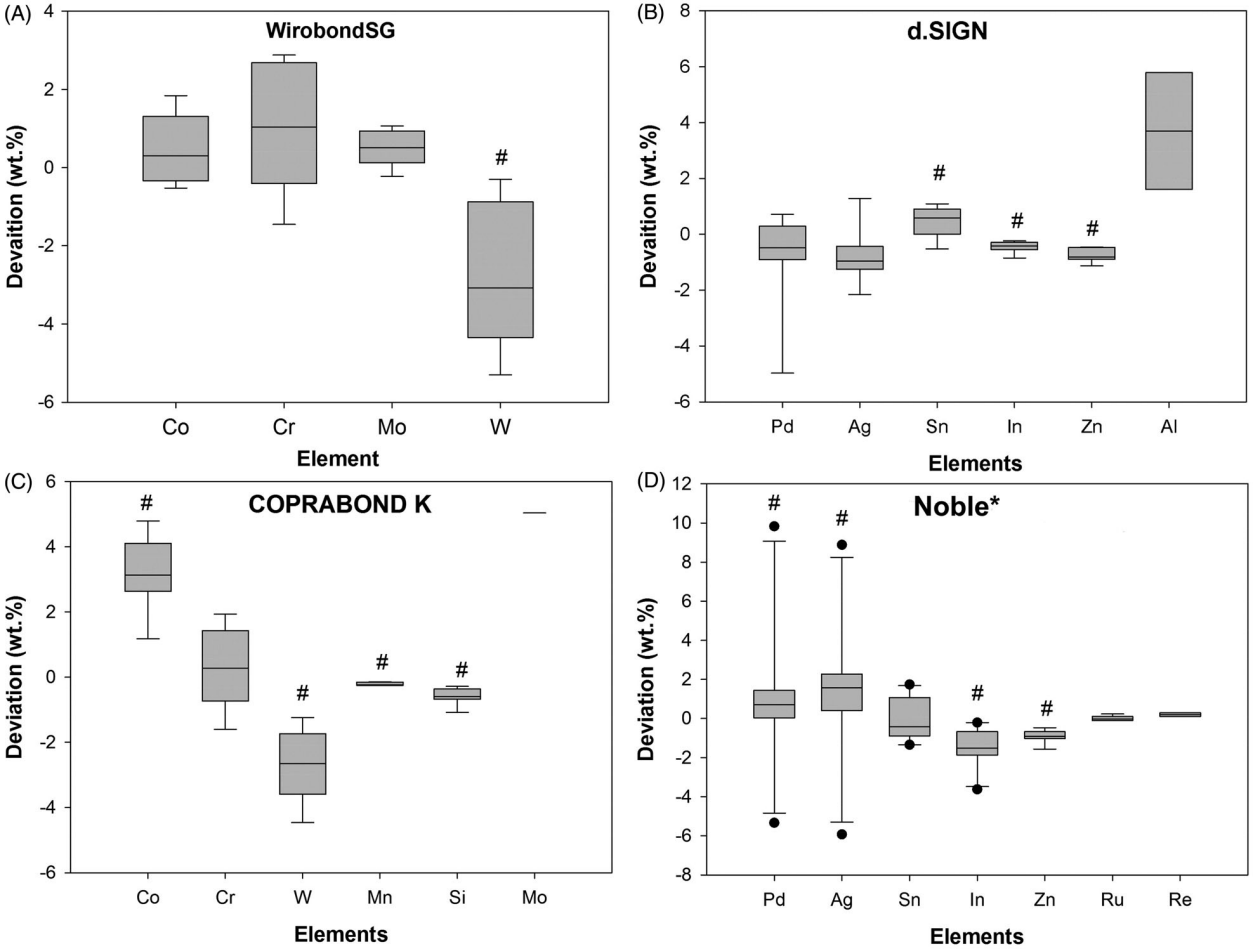
Whisker diagrams depicting distribution of data concerning alloy content deviation. Alloys ‘Noble’ (D) had large variations, hence the outliers in several of the constituents, whereas d.SIGN53 (B) showed little variation. *Noble metal diagram includes both Noble metal 1 and 2; these two alloys are both from Lab D and were termed Noble metal, however with slight differences in wt. % of each element. #statistically significant difference between the mean of the sampled population and the hypothesized population mean (*p* < .05).

In total, 62 PFMs were analyzed; 21 imported and 41 nonimported. 32 crowns were of noble metal composition and 30 were predominantly base metal. Laboratory A delivered 22 crowns, B delivered 10 crowns, C delivered 9 crowns, D delivered 18 crowns, E, F and G delivered 1 crown each. The analysis revealed that, within the limitations of the testing method and sample selection, a majority of crowns had small or large deviations (Figure 1 and Table 3). In general, large deviations were more common in imported compared to nonimported crowns. However, nonimported crowns displayed a higher occurrence of small deviations and unspecified alloys. In addition, own-brand-label (OBL) alloys showed a larger variation in alloy content (Figure 2), Lab D labelled these PFMs as noble metal with specified content, in comparison to branded alloys.

**Table 3.** Distribution and number of base-metal crowns sorted in categories according to degree of deviation.

Alloy crown type	Distribution (%)	
**All crown alloys**	**Imported (*n* = 21)**	**Nonimported (*n* = 41)**
No deviation	19 (*n* = 4)	15 (*n* = 6)
Small deviation	14 (*n* = 3)	32 (*n* = 13)
Large deviation	43 (*n* = 9)	20 (*n* = 8)
Incorrect alloy	N/A	2 (*n* = 1)
Unspecified alloy	24 (*n* = 5)	32 (*n* = 13)
**Precious crown alloy**	**Imported (*n* = 12)**	**Nonimported (*n* = 20)**
No deviation	25 (*n* = 3)	25 (*n* = 5)
Small deviation	N/A	30 (*n* = 6)
Large deviation	33 (*n* = 4)	25 (*n* = 5)
Incorrect alloy	N/A	N/A
Unspecified alloy	42 (*n* = 5)	20 (*n* = 4)
**Non-precious crown alloy**	**Imported (*n* = 9)**	**Nonimported (*n* = 21)**
No deviation	11 (*n* = 1)	5 (*n* = 1)
Small deviation	33 (*n* = 3)	33 (*n* = 7)
Large deviation	56 (*n* = 5)	14 (*n* = 3)
Incorrect alloy	N/A	5 (*n* = 1)
Unspecified alloy	N/A	43 (*n* = 9)
**Category**	**Definition**	
No deviation	Minor deviations in major constituents (elements that comprises >20 wt.%) and slight deviations in additional elements that comprise <10 wt.%.	
Small deviation	<5 wt.% in regard to major constituents and/or deviations >1 wt.% in additional elements that comprise <10 wt.%.	
Large deviation	>5wt.% in regards to major constituents and missing and/or additional elements.	
Incorrect alloy	Crowns that comprised of elements similar to a different type of alloy. For example a crown marked as CopraBond K with results showing elements such as silver (Ag) and palladium (Pd).	
Unspecified alloy	Crowns delivered without or with lacking alloy information.	

Only 1 crown (crown no. 9, nonimported) was delivered with an incorrect documented alloy. The crown was originally ordered as a predominantly base metal Co–Cr-based crown, however documentation from the laboratory specified a Cara SLM alloy. Analysis resulted that the substructure comprised of an Ag–Pd based alloy (56 wt. % Pd and 37 wt.% Ag), a composition similar to Argelite61. The most common deviation in CopraBond K (Table S.06) alloys leading to crowns being placed in the category large deviation (*p* < .05) was due to tungsten (W) content, where a mean reduction of 2.7 wt. % was observed. Smaller deviations for cobalt (C) (*p* < .05) and chromium (Cr) were also found. There was a significant deviance from the mean also for elements Co, W. Mn and Si (Figure 2(C)). Three Argelite 61 crowns (crowns no. 3, 48 and 49, imported) were placed in the large deviation category as they had large and significant differences for Ag, Pd and additional elements (Table S.03 and Table S.05). The common significant deviation was increased Ag and reduced Pd, respectively 15 wt. % and 12 wt. %. Other significant deviations were found for W in WirobondSG (Figure 1(A)), Sn and In for d.Sign alloys (Figure 2(B)).

**Figure 3.**
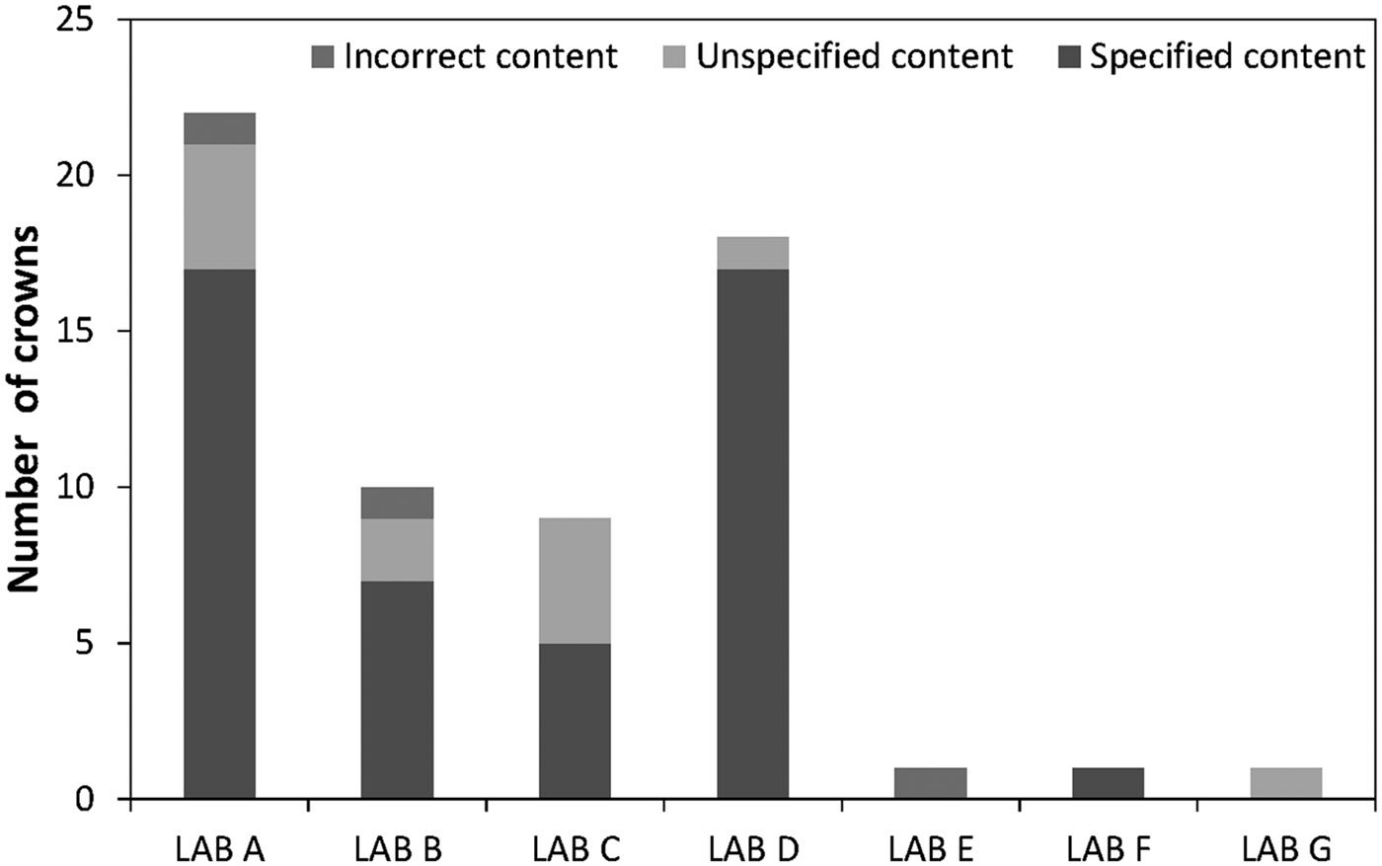
Information provided by the seven different dental laboratories showing level of ‘incorrect content’, ‘unspecified content’ and ‘specified content’.

Four crowns contained aluminum (Al) (crowns no. 23 and 46, imported, no. 24 and 34, nonimported). This element was not specified in the accompanying documentation, nor does it belong in the relevant branded alloys: Argelite61, Noble metal or Remanium 2001 (Table S.03 and S.06). In total, 18 PFMs were delivered without or with lacking information, and there were variations in enclosed content information from the laboratories (Figure 3).

## Discussion

The null hypothesis that there was that there would be no difference in the level of compositional discrepancy between imported and non-imported PFMs. Nonetheless, the results from the present study show that patients risk receiving a crown with deviating alloy content regardless whether the crown had been produced at a Norwegian dental laboratory or had been imported. Several key metallic elements, which are important for both porcelain fusing, strength and casting of metal-ceramic crowns, deviated significantly in the tested crowns. Therefore, it is expected that these crowns´ survival time will be affected, particular in respect to ceramic chipping [10–12]. There were no obvious reasons for the observed deviations; possible scenarios could be casting contaminations, reuse of casted metal or human error. There is obviously a clear need for improved quality control between laboratory and dentist as there was generally a lack of declarations from most of the laboratories.

Despite large deviations in several crowns, analysis of the metal alloy compositions from Norwegian and international dental laboratories generally showed decent agreement in accordance with ISO 22674: 2006 Dentistry – Metallic materials for fixed and removable restorations and appliances. The ISO standard (ISO 22674) sets limits for maximum content of Cd, Ni and Be. These three elements are potential allergens or may be the source of hazardous gases during production [13–15]. None of the analyzed crowns contained more than 0.02% of Cd or more than 0.1% of Ni. Be cannot be traced precisely with the Energy-dispersive X-ray spectroscopy (EDX-SEM), as EDX-SEM element identification area from B5 to Am95 in the periodic table [16].

Sixty-two crowns were analyzed: 44 with specified alloy content and 18 without or with lacking information. A deviance >5wt.% outside the normal range as well as specimens that contained foreign elements were deemed to contain a deviation in this study. Thirteen of the 44 specified crowns had a deviation larger than 5 wt.% or foreign elements such as aluminum (Al). A general consistency between the specified and analyzed alloy content was determined for 70% of the crowns. However, metal alloys used in PFMs constructions often contain elements that only make up smaller parts of the total wt.%, such as alloying and oxide elements (Tables 1 and 2). As these elements play an important role in the substructures´ properties they are discussed in the following paragraphs, with descriptions of potential clinical affects. Traditional alloys for PFMs were gold based with varying percentage of gold. These are specified according to gold content [17]. Although nongold noble alloys are currently more frequently used due to their low cost [18], high-gold-based alloys are still used in many private practices and institutions.

In alloys based on cobalt (Co) and chromium (Cr) such as CopraBond K, Co is the source of stiffness, strength and hardness, whereas Cr provides corrosion resistance [19]. However, Cr contents >30% makes casting difficult as well as making the alloy more brittle [20]. Of the 62 analyzed crowns, only one Remanium 2001 crown had >30% Cr content (crown no. 57 with 30.86 wt. % Cr). A low wt.% Cr would suggest that the crown will experience early signs of wear due to corrosion.[21]

Molybdenum (Mo) is an element found in Co–Cr alloys used to both strengthen [22] and lower the expansion coefficient. A content of between 3–6% Mo increases the strength [23,24], whilst having too little Mo would suggest lower strength. Only one Co–Cr crown (crown. no 35) had no traces of Mo suggesting lower strength and possible additional strain on the outer porcelain veneer due to a higher expansion coefficient. Other elements such as tin (Si) and manganese (Mn) increase flowability and castability. Tungsten (W) and carbon (C) strengthen the alloy [25,26]. A common trait in many crowns was a reduction of W weight percentage suggesting larger risks of technical faults such as casting irregularities during crown production, which in turn could affect clinical life span [27].

Nongold containing noble alloys consist mostly of silver (Ag), palladium (Pd) or platinum (Pt). Alongside these major elements copper (Cu) can be added to increase hardness and strength [28]; however, this gives the alloy a reddish color. Tin (Sn) lowers the melting interval in palladium alloys and works well when soldered in addition to improving castability and cures cast-gold alloys. Only one crown contained Cu (2.1 wt. %). Four crowns (crown no. 3, 20, 48 and 49) had a high content of silver (Ag) that is likely to cause greenish-yellow discoloration. Three of these crowns were imported and produced by Laboratory D. The deviation in both crowns was 9.1 wt. % Ag and decrease of 10.7 wt. % Pd. Ag is used in Pd-based alloys to increase the thermal expansion coefficient [29] and to form solid solution which hardens the alloy [30,31]. Crowns 48 and 49 (both imported) were especially alarming with an increase of 18.4 wt. % and 20.7 wt.% Ag respectively.

Indium (In) and gallium (Ga) create oxides for binding to the veneering layer. Additionally, Indium makes high-gold alloys harder, and gallium increases yield strength for palladium alloys [32,33]. Binding oxides is of outmost importance in PFMs as the esthetic porcelain veneer must be able to sufficiently bond to the metal substructure. Insufficient binding yields a higher risk for chipping and porcelain fracture. Most metal-alloys in this study that included In were supposed to have values ranging between 1–7 wt.%; however, four crowns (crown no. 15 and 20 nonimported, 3 and 44 imported) had <1 wt.% In, where two had no trace of this element. Several crowns had lower content amounts of either In and/or Ga making this a likely contributor to future porcelain fractures due to reduced bonding between metal and ceramic [34,35]. Iridium (Ir), rhodium (Rh) and ruthenium (Ru) are all grain refiners, and it is only with these elements that one can harden an alloy without decreasing ductility [35]. Small traces of these elements were found in several crowns, and due to the low wt. %, these were mostly disregarded due to the limitations of analysis method. Since key metallic elements (W, In, Sn Pd, Ag), which are imperative for PFMs performance, deviated significantly on the tested crowns, it is expected that survival time of these crowns will be affected. Even though the analysis were only performed on dental crowns produced by Norwegian dental laboratories, it is expected that similar findings would occur in other countries

Some crowns contained aluminum (Al). Al is used in Ni-Cr alloys, as it increases both tensile and yield strength by forming Ni3Al (metal oxide) [36,37]. Al, however, is an element not included in neither Noble metal 1 nor 2, d.SIGN®53 nor Remanium 2001 alloys. It is unclear why a relatively high level of Al is present in the noble metal alloys. Two crowns were delivered with noble metal alloy content with silver (Ag) and palladium (Pd) as its main constituents. In one case, the crown was delivered with misleading information stating that it was a Cara SLM alloy, and in the second case, it was unspecified. Although allergies toward palladium are uncommon, the prevalence of contact allergic reactions toward this alloy–metal had been reported to be 7.4% in dental patients [38]. Pd-based alloys are stated to be the cause of some cases of stomatitis [39] and oral lichenoid reactions [40]. An Austrian study found that 8.3% of 1382 patients with eczema were sensitive to Pd [41]. Furthermore, allergy to Pd is reported to have occurred in patients who are sensitive to Ni [42–44]. Ni is the most allergic metal known, with an incidence of 10–20% allergic reactions [43]. Reactions to Ni are more common among woman, presumably due of chronic exposure to Ni-containing jewelry [43]. A dentist makes the choice of metal-alloy in collaboration with the patient and is based on several factors including known allergies. Even with a detailed medical history and correct choice of metal-alloy, these patients could unknowingly receive a custom fit medical device with a metal causing an allergic reaction.

Approximately 50% of both imported and nonimported crowns had large discrepancies between the specified and analyzed alloy content. However, due to the uneven number in the two groups (41 nonimported, 21 imported), this equated to 43% and 20% in the imported and nonimported respectively (Table 3). This is an indicator that deviations in alloy content are more common amongst imported crowns. This finding is with agreement to the study conducted by the Swedish Medical Products Agency (Läkemedelsverket) resulting in imported crowns having a higher occurrence of incorrect specified alloy content in comparison to those produced in Sweden [7]. Figure 1 shows the differences between imported and nonimported PFMs.

Several crowns had descriptive information limited to base metal/noble metal, however according to the European Medical Devices Directive (MDD) metal–alloy information used in dental crowns must accompany PFMs as these are classified as individualized equipment. Figure 3 illustrates a comparison of specified content, no content information and incorrect information where one could deem incorrect information to include specimens with contradicting alloys, for example, ordered Co-Cr and received Ag-Pd.

## Conclusion

Our results indicate that there is a compositional discrepancy in alloy content between imported and non-imported crowns, with imported crowns being more likely to have large alloy content deviations. Both imported and non-imported crowns were just as likely to provide specified content information. Several crowns were found to deviate from their presumed composition. In addition, branded alloys were more reliable regarding its alloy composition when compared to ‘own-brand-label’ alloys. A majority of PFMs only had smaller negligible deviations between labelled and analyzed composition. However, some key metallic elements (W, In, Sn Pd, Ag), which are imperative for PFMs performance, deviated significantly on the tested crowns.

## Supplementary Material

Supplemental Material
