# Supplementary material for: Discrepancy in alloy composition of imported and non-imported porcelain-fused-to-metal (PFM) crowns produced by Norwegian dental laboratories
Source: Biomater Investig Dent. 2020 Feb 11;7(1):41–9. doi: 10.1080/26415275.2020.1724512 (PMC7033715; doi:10.1080/26415275.2020.1724512)
Supplement: Supplemental Material [file IABO_A_1724512_SM6680.zip › Table S05.docx]

|  | Precious | d.SIGN®53 | Argelite 61 |  |
| --- | --- | --- | --- | --- |
| Elements | **Concentration (wt. %)** | | | **Primary role of element** |
| Pd | 61.4 | 53.8 | 60.6 | Main component |
| Ag | 26.0 | 34.9 | 28.1 | Increase the thermal expansion coefficient |
| Sn | 6.0 | 7.7 | 2.5 | Lowers the melting interval, improving castability |
| In | 4.0 | 1.7 | 6.6 | Create oxides for binding to the veneering layer |
| Sn | 2.5 | 1.2 |  | Lower melting point |
| Ru | 0.1 | < 1.0 | < 1.0 | grain refiners |
| Re |  | < 1.0 | < 1.0 | grain refiners |
| Li |  | < 1.0 |  | grain refiners |
| Pt |  | < 1.0 |  |  |
| Ga |  |  | 2.1 | Create oxides for binding to the veneering layer |

**Table S 05** The individual components and the desired concentrations content of various branded noble alloys used in the study
