# Supplementary material for: Discrepancy in alloy composition of imported and non-imported porcelain-fused-to-metal (PFM) crowns produced by Norwegian dental laboratories
Source: Biomater Investig Dent. 2020 Feb 11;7(1):41–9. doi: 10.1080/26415275.2020.1724512 (PMC7033715; doi:10.1080/26415275.2020.1724512)
Supplement: Supplemental Material [file IABO_A_1724512_SM6680.zip › Table S06.docx]

|  | CopraBond K | Cara SLM | Kera® (milled) | Remanium2001 |  |
| --- | --- | --- | --- | --- | --- |
| Elements | **Concentration (wt. %)** | | | | **Primary role of element** |
| Co | 61.0 | 61.8 – 65.8 | 61.7 | 63.0 | Stiffness, strength and hardness |
| Cr | 28.0 | 23.7 – 25.7 | 27.8 | 23.0 | Corrosion resistance |
| Mo |  | 4.6 – 5.6 |  | 7.3 | Strength, lower the expansion coefficient |
| W | 8.5 | 4.9 – 5.9 | 8.5 | 4.3 | Strength |
| Mn | 0.3 | < 0.1 | 0.3 | <1.0 | Increase flowability and castability |
| Fe | < 0.5 | < 0.5 | 0.2 |  | Strengthen effect at high temperature |
| Si | 1.7 | < 1.2 | 1.6 | 1.6 |  |

**Table S 06** The individual components and the desired concentrations content of various branded base metal alloys used in the study
