# Supplementary material for: Discrepancy in alloy composition of imported and non-imported porcelain-fused-to-metal (PFM) crowns produced by Norwegian dental laboratories
Source: Biomater Investig Dent. 2020 Feb 11;7(1):41–9. doi: 10.1080/26415275.2020.1724512 (PMC7033715; doi:10.1080/26415275.2020.1724512)
Supplement: Supplemental Material [file IABO_A_1724512_SM6680.zip › Table S01.docx]

| **Lab** | Crown | Label | **Pd** | **Ag** | **Sn^#^** | **In^#^** | **Ru** | **Al** | **Ga** |  |
| --- | --- | --- | --- | --- | --- | --- | --- | --- | --- | --- |
| **A** | 28 | X |  |  |  |  |  |  |  |  |
|  | 30 | d.SI | 0.7 | -0.6 | 0.4 | -0.5 |  |  |  |  |
|  | 32 | d.SI | 0.1 | -1.2 | 0.6 | -0.4 |  |  |  |  |
| **B** | 44 | d.SI | 0.3 | -0.3 | 0.9 | -0.9 |  |  |  |  |
| **D** | 3 | Arg 61 | -5.9 | 8.1 | 4.6 | -6.6 |  |  | -1.8 |  |
|  | 23 | P_1_ | -2.7 | -7.9 | -3.0 | -1.7 | -0.10 | 9.0 |  |  |
|  | 47 | X |  |  |  |  |  |  |  |  |
|  | 48 | Arg 61 | -15.1 | 18.4 | -2.5 | -0.2 |  |  | -1.8 |  |
|  | 49 | Arg 61 | -16.4 | 20.7 | -2.5 | -2.4 |  |  | -2.0 |  |
| **C** | 39 | X |  |  |  |  |  |  |  |  |
| **G** | 36 | X |  |  |  |  |  |  |  |  |
| **E** | 38 | Z |  |  |  |  |  |  |  |  |
| **Table S1:** Imported Crowns – Noble metal alloys. Z: Labelled as both CoCr and Noble metal. Analysis revealed noble metal composition. One Imported noble metal crown was found to contain Al, an element not included in the enclosed alloy description. #Statistically significant difference between the mean of the sampled population and the hypothesized population mean (p<0.05). Abbreviations: d.SI (d.SIGN), Arg 61 (Argelite 61), P (Noble metal 1 and 2). Empty box: amount below detection limit. | | | | | | | | | | |
