# Supplementary material for: Discrepancy in alloy composition of imported and non-imported porcelain-fused-to-metal (PFM) crowns produced by Norwegian dental laboratories
Source: Biomater Investig Dent. 2020 Feb 11;7(1):41–9. doi: 10.1080/26415275.2020.1724512 (PMC7033715; doi:10.1080/26415275.2020.1724512)
Supplement: Supplemental Material [file IABO_A_1724512_SM6680.zip › Table S02.docx]

| **Lab** | Crown | Label | **Co** | **Cr** | **W^#^** | **Mn^#^** | **Fe** | **Si** | **Mo** | **Ga** |  |
| --- | --- | --- | --- | --- | --- | --- | --- | --- | --- | --- | --- |
| **A** | 1 | CB K | 3.1 | -0.8 | -1.2 | -0.3 |  | -0.6 |  |  |  |
|  | 2 | CB K | 3.7 | 0.3 | -2.8 | -0.3 |  | -0.6 |  |  |  |
|  | 6 | CB K | 4.8 | 0.3 | -3.9 | -0.2 |  | -0.3 |  |  |  |
|  | 8 | CB K | 4.5 | -0.7 | -2.7 | -0.1 |  | -0.6 |  |  |  |
|  | 12 | CB K | 2.9 | 1.4 | -3.2 | -0.2 |  | -0.4 |  |  |  |
|  | 21 | CB K | 2.4 | 1.4 | -2.5 | -0.2 |  | -0.5 |  |  |  |
|  | 22 | X |  |  |  |  |  |  |  |  |  |
|  | 35 | X |  |  |  |  |  |  |  |  |  |
| **B** | 10 | CB K | 3.4 | -0.6 | -1.3 | -0.2 |  | -0.7 |  |  |  |
|  | 16 | CB K | 1.9 | 1.9 | -2.1 | -0.1 |  | -0.3 |  |  |  |
|  | 17 | CB K | 2.9 | -1.6 | -4.5 | -0.3 |  | -1.1 | 5.0 |  |  |
|  | 9 | C SLM | * |  |  |  |  |  |  |  |  |
|  | 18 | K®D | 3.9 | -1.1 | -2.2 | -0.1 | -0.2 | -0.3 |  |  |  |
|  | 51 | X |  |  |  |  |  |  |  |  |  |
|  | 52 | W280 | 2.7 | 2.4 |  |  |  |  | -2.6 | -2.6 |  |
| **C** | 42 | X |  |  |  |  |  |  |  |  |  |
|  | 43 | X |  |  |  |  |  |  |  |  |  |
|  | 53 | X |  |  |  |  |  |  |  |  |  |
|  | 58 | X |  |  |  |  |  |  |  |  |  |
|  | 61 | X |  |  |  |  |  |  |  |  |  |
|  | 62 | X |  |  |  |  |  |  |  |  |  |
| **Table S 02: Non-imported Produced Crowns – Predominantly base metal alloys** (CoCr) (n=21) * Labelled as noble metal alloy (Cara SLM), but analysis revealed no noble metal composition. One Non-imported predominantly base metal crown was found to contain Mo, an element not included in the enclosed alloy description. Abbreviations: CB K (*Coprabond K*), C SLM (*Cara SLM*), K®D (*Kera®-disc),* W280 (*Wirobond 280*). ^#^Statistically significant difference between the mean of the sampled population and the hypothesized population mean (p<0.05). Empty box: amount below detection limit. | | | | | | | | | | | |
