# Supplementary material for: Discrepancy in alloy composition of imported and non-imported porcelain-fused-to-metal (PFM) crowns produced by Norwegian dental laboratories
Source: Biomater Investig Dent. 2020 Feb 11;7(1):41–9. doi: 10.1080/26415275.2020.1724512 (PMC7033715; doi:10.1080/26415275.2020.1724512)
Supplement: Supplemental Material [file IABO_A_1724512_SM6680.zip › Table S03.docx]

| **Lab** | Crown | Label | **Co** | **Cr** | **W** | **Si** | **Mo** | **Al** |  |
| --- | --- | --- | --- | --- | --- | --- | --- | --- | --- |
| **A** | 54 | WSG | 0.8 | -1.5 | -1.5 |  | 0.5 |  |  |
|  | 55 | WSG | -0.2 | 2.5 | -3.4 |  | 1.1 |  |  |
|  | 56 | WSG | 1.8 | 1.0 | -5.3^#^ | 2.9 | 0.5 |  |  |
|  | 59 | WSG | 0.3 | 0.6 | -0.3^#^ |  | -0.2 |  |  |
|  | 60 | WSG | -0.5 | 2.8 | -3.1 |  | 0.8 |  |  |
| **D** | 46 | R2001 | -0.8 | 4.6^#^ |  |  | -0.7 | 3.6 |  |
|  | 50 | R2001 | -0.1 | 5.3^#^ | -4.3 | 0.9 | -1.0 |  |  |
|  | 57 | R2001 | -3.3^#^ | 2.9^#^ | -4.3 | 0.9 | -0.5 |  |  |
| **F** | 37 | CoCr | 2.9 | 2.6 |  |  | 0.6 |  |  |
| **Table S 03:** Imported Crowns – Predominantly base metal alloys (CoCr) (n=9). CoCr: Delivered with enclosed specification on alloy composition, thus enabled analysis comparison. Abbreviations: WSG (Wirobond SG), R2001 (Remanium 2001). #Statistically significant difference between the mean of the sampled population and the hypothesized population mean (p<0.05). Empty box: amount below detection limit. | | | | | | | | | |
