# Supplementary material for: Discrepancy in alloy composition of imported and non-imported porcelain-fused-to-metal (PFM) crowns produced by Norwegian dental laboratories
Source: Biomater Investig Dent. 2020 Feb 11;7(1):41–9. doi: 10.1080/26415275.2020.1724512 (PMC7033715; doi:10.1080/26415275.2020.1724512)
Supplement: Supplemental Material [file IABO_A_1724512_SM6680.zip › Table S04.docx]

| **Lab** | Crown | Label | **Pd^#^** | **Ag^#^** | **Sn** | **In^#^** | **Zn^#^** | **Ru** | **Re** | **Al** | **Au** |
| --- | --- | --- | --- | --- | --- | --- | --- | --- | --- | --- | --- |
| **A** | 26 | d.SI | -0.9 | -0.9 | 0.7 | -0.3 | -0.9 |  |  |  |  |
|  | 27 | d.SI | -0.5 | 1.3 | -0.5 | -0.4 | 0.9 |  |  |  |  |
|  | 29 | d.SI | 0.3 | -1.3 | 0.8 | -0.2 | -0.7 |  |  |  |  |
|  | 33 | d.SI | -0.9 | -1.1 | 1.1 | -0.3 | 1.1 |  |  |  |  |
|  | 34 | d.SI | -0.5 | -0.5 | 0 | -0.5 | -0.9 |  |  | 1.6 |  |
|  | 31 | X |  |  |  |  |  |  |  |  |  |
| **B** | 24 | d.SI | -4.1 | -2.1 |  | 0.6 | -0.5 |  |  | 5.8 |  |
|  | 45 | X |  |  |  |  |  |  |  |  |  |
| **D** | 4 | P_1_ | 0.4 | 0.4 | 0.6 | -0.8 | -0.6 | -0.1 |  |  |  |
|  | 5 | P_1_ | 2.2 | 2.1 | -1.4 | -2.1 | -1.0 | - | 0.1 |  |  |
|  | 7 | P_1_ | 1.1 | 1.9 | -0.7 | -1.6 | -0.9 | 0.1 |  |  |  |
|  | 11 | P_1_ | 9.8 | -5.9 | -1.3 | -1.8 | -1.0 | -0.1 | 0.3 |  |  |
|  | 13 | P_1_ | 0.3 | 2.6 | -0.7 | -1.7 | -0.5 |  |  |  |  |
|  | 14 | P_1_ | 1.1 | 0.7 | -0.2 | -0.9 | -0.8 |  |  |  |  |
|  | 15 | P_2_ | 1.4 | 1.2 | -0.2 | -1.0 |  |  |  |  | -0.3 |
|  | 19 | P_1_ | 1.0 | 1.7 | -0.6 | -1.4 | -0.9 | 0.2 |  |  |  |
|  | 20 | P_1_ | -5.3 | 8.9 | 1.7 | -3.6 | -1.6 |  |  |  |  |
|  | 25 | P_1_ | 0.2 | 0.4 | -0.2 | 1.0 | -0.2 |  |  |  |  |
| **C** | 40 | X |  |  |  |  |  |  |  |  |  |
|  | 41 | X |  |  |  |  |  |  |  |  |  |

**Table S 04:** Non-imported Produced Crowns – Noble metal alloys (n=20). Two Non-imported noble metal crowns were found to contain Al, an element not included in the enclosed alloy description. . #Statistically significant difference between the mean of the sampled population and the hypothesized population mean (p<0.05). Abbreviations: d.SI (d.SIGN), P (Noble metal 1 and 2). #Statistically significant difference between the mean of the sampled population and the hypothesized population mean (p<0.05). Empty box: amount below detection limit.
